# Evaluating the Disparate Use of Knee Arthroplasty Among Minorities Using Social Vulnerability Index

**DOI:** 10.1016/j.artd.2025.101702

**Published:** 2025-06-09

**Authors:** Manasa Pagadala, Rachel Bergman, T. Jacob Selph, Patricia Franklin, Adam I. Edelstein, Linda I. Suleiman

**Affiliations:** a Northwestern University Feinberg School of Medicine, Chicago, IL, USA; b Division of Orthopaedic Surgery, Northwestern University, Chicago, IL, USA

**Keywords:** Social vulnerability index, Total knee arthroplasty, Healthcare equity, Osteoarthritis

## Abstract

**Background:**

Total knee arthroplasty (TKA) is an effective treatment for advanced osteoarthritis, but disparities in its utilization exist, particularly by gender, race, socioeconomic status, and geography. Social determinants of health may contribute to these disparities. This study examines the relationship among the Social Vulnerability Index (SVI), a measure of social determinants of health, and the likelihood of receiving a surgeon's recommendation for TKA.

**Methods:**

This prospective, observational study included 314 patients with primary knee osteoarthritis deemed “appropriate” for TKA based on the American Academy of Orthopaedic Surgeons Appropriate Use Criteria. Patients were recruited from 4 fellowship-trained arthroplasty surgeons at a single academic hospital in Chicago. The primary outcome was whether patients received a surgeon recommendation for TKA, analyzed in relation to their SVI. Bivariate and multivariable analyses were performed, adjusting for patient demographics, body mass index, Charlson Comorbidity Index, and surgeon factors.

**Results:**

Of the 314 patients appropriate for TKA by Appropriate Use Criteria guidelines, 39.2% received a surgeon recommendation. Patients with lower SVI scores were more likely to receive a recommendation (0.6596 vs 0.7556, *P* = .284). Multivariable analysis showed that lower SVI scores were independently associated with higher odds of a surgeon recommendation (odds ratio [OR]: 0.705, *P* = .028), particularly in the subcategories of socioeconomic status (OR: 0.754, *P* = .034) and household characteristics (OR: 0.803, *P* = .049).

**Conclusions:**

Lower SVI scores are associated with increased likelihood of receiving a TKA recommendation, highlighting the impact of social vulnerability on clinical decision-making and the need for further investigation into mitigating biases in surgical decisions.

## Introduction

Osteoarthritis (OA) is the most prevalent joint disease and a leading source of chronic pain and disability in the United States [1]. Nationally, it is predicted to affect nearly 78.4 million or 26% of adults by 2040 [2]. For patients who fail conservative management, surgical management with total knee arthroplasty (TKA) is known to alleviate pain and improve joint function and overall quality of life. Despite national efforts, there remains a frank disparity in utilization of TKA with respect to gender, race, socioeconomic status, and geography [3].

Social determinants of health (SDOH) such as health insurance, socioeconomic status, access to care, and education are proposed factors contributing to disparities in care. Using the Centers for Disease Control Social Vulnerability Index (SVI), which assigns scores based on 16 societal variables across 4 categories—socioeconomic elements, household characteristics, racial and ethnic minority status, and housing and transportation—previous research has linked higher SVI scores to extended hospital stays and a greater probability of nonhome discharges after TKA [4]. Considering the growing recognition of social determinants in healthcare disparities, the specific impact of these factors on utilization of TKA by patients is not well understood.

Studies in nonorthopaedic fields have found that more vulnerable patients are less likely to undergo elective surgical hernia repair, even after adjusting for demographics, insurance, and urbanicity [5]. Within orthopaedics, certain studies have explored other indices similar to SVI: Hartnett et al demonstrated that those with higher levels of social deprivation measured by Social Deprivation Index (SDI) were less likely to undergo TKA [6]. To our knowledge, the present study is the first to explore whether SVI is associated with a difference in American Academy of Orthopaedic Surgeons (AAOS) TKA Appropriate Use Criteria (AUC) rating and individual surgeon recommendations to undergo TKA. TKA utilization is largely governed by both surgeons' offer of the procedure as well as patients' willingness to pursue surgical management [7]. Therefore, we sought to understand the influence of patient social vulnerability on a surgeon's recommendations given that potential biases—whether implicit or explicit—may influence the surgeon's recommendations. With this goal, we conducted a prospective, observational study to determine whether SVI is associated with a difference in surgeon likelihood to recommend TKA.

## Material and methods

### Study population

Patients were recruited from orthopaedic surgery clinics of 4 fellowship-trained arthroplasty surgeons (1 Black, 3 Non-Hispanic White [NHW]) at a single academic hospital in Chicago, Illinois. Patients aged ≥18 years who presented for management of primary knee OA between January 30, 2023 and April 25, 2023 met inclusion criteria and were considered eligible for inclusion in final analysis. There were 402 consecutive patients who presented for management of knee OA to the clinics of 4 fellowship-trained arthroplasty surgeons at a single academic center, 314 of which were “appropriate” for TKA and included in final analysis (Table 1). Surgeon #1 self-identified as Black, while Surgeons #2, #3, and #4 self-identified as NHW (Table 2).

**Table 1:** Patients by level of appropriateness for 3 treatment categories in knee osteoarthritis: surgical management appropriate.

Osteoarthritis of the knee: surgical management appropriate use criteria categories	TKA	UKA	Realignment osteotomy
Appropriate	314	13	0
May be appropriate	88	126	23
Rarely appropriate	0	263	379

TKA, total knee arthroplasty; UKA, unicompartmental knee arthroplasty.

**Table 2:** Demographic data, BMI, and CCI with breakdown by surgeon.^a^

Level of appropriateness for TKA	Surgeon recommended TKA? (N = 123)	Race/ethnicity											Insurance				Sex		Age	BMI	CCI
		NHW	Black	Asian	Native American	Hawaiian/Pacific Islander	Hispanic ethnicity, race: “Other”	Hispanic ethnicity, Race: White	Hispanic ethnicity, Race: Black	Other	Declined	Unknown	Medicare	Medicaid	Private	Other	Men	Women			
Appropriate (N = 314)																					
Surgeon #1 (N = 120)	69 (57.5%)	22 (31.9%)	33 (47.8%)	3 (4.34%)	1 (1.44%)	0	7 (10.1%)	1 (1.44%)	0	0	1 (1.44%)	1	34 (49.3%)	4 (5.80%)	30 (43.5%)	1 (1.44%)	25 (36.2%)	44 (63.8%)	67.46 (10.46)	30.47 (7.35)	3.12 (2.08)
Surgeon #2 (N = 92)	23 (25.0%)	16 (69.6%)	6 (26.1%)	0	0	0	1 (4.34%)	**0**	0	0	0	0	13 (56.5%)	0	10 (43.4%)	0	10 (43.4%)	13 (56.5%)	68.13 (10.54)	32.84 (7.35)	3.57 (2.09)
Surgeon #3 (N = 37)	7 (18.9%)	3 (42.9%)	1 (14.3%)	0	0	0	0	1 (14.3%)	0	0	1 (14.3%)	1 (14.3%)	1 (14.3%)	1 (14.3%)	5 (71.4%)	0	1 (14.3%)	6 (85.7%)	57.57 (9.86)	33.73 (7.40)	2.14 (2.08)
Surgeon #4 (N = 65)	24 (36.9%)	13 (54.2%)	8 (33.3%)	0	0	0	2 (8.33%)	0	0	0	0	1 (4.17%)	13 (54.2%)	5 (20.8%)	5 (20.8%)	1 (4.17%)	7 (29.2%)	17 (70.8%)	65.88 (10.51)	31.61 (7.32)	4.04 (2.07)

BMI, body mass index; CCI, Charlson Comorbidity Index; NHW, Non-Hispanic White; TKA, total knee arthroplasty.

Surgeon #1 self-identified as Black, while Surgeons #2, #3, and #4 self-identified as NHW.

Mean and standard deviation reported for age, BMI, and CCI.

a % is percentage of that surgeon's patient N in that appropriateness category.

### Data collection

This study was institutional review board–approved through Northwestern University. Patient clinical and demographic data were input into the AAOS AUC entitled “Osteoarthritis of the Knee: Surgical Management” to generate a guideline-based procedure recommendation for TKA [1]. To minimize selection bias, only the 314 patients considered “appropriate” for TKA were included in final analysis (Table 1). Thirteen patients were considered “appropriate” for unicompartmental knee arthroplasty and 0 were “appropriate” for realignment osteotomy and were excluded from final analysis (Table 1). Patients' medical records were reviewed to determine whether the orthopaedic surgeon had recommended TKA and obtain sociodemographic data (self-identified patient race/ethnicity, age, gender, body mass index [BMI], insurance type, smoking status, Charlson Comorbidity Index [CCI], and ZIP code). Surgeon race/ethnicity was also recorded at the time of clinic visit.

### Study outcome variables

Of the 402 patients who presented to clinic for primary knee OA, 314 patients were considered “appropriate” for TKA and the remaining 88 were excluded as they did not meet this criterion. The primary outcome was whether patients received a recommendation for TKA from the treating orthopaedic surgeon. This was ascertained by trained research staff who reviewed the orthopaedic visit note in each patient's electronic medical record to determine whether TKA was documented as the recommended treatment. Patients without a documented visit and surgeon recommendation in their electronic medical record were excluded from analysis. No patients were excluded from analysis as they all had a documented visit and surgeon recommendation.

### Study instruments

The primary study instrument used was the AAOS AUC entitled “Osteoarthritis of the Knee: Surgical Management.” This AUC is accompanied by several assumptions that surgeons should consider prior to utilization to ensure that the criteria are employed in the correct clinical setting (ie, adults who have been diagnosed with primary OA of the knee who have failed nonoperative treatments [8]. The AUC guidelines and accompanying assumptions are described in the Supplementary Material, and a web-based application can be found at www.orthoguidelines.org/auc [9,10] to help clinicians develop a plan for managing knee OA. These guidelines serve to address surgeon questions regarding the use of TKA, unicompartmental knee arthroplasty, and local osteotomy at the level of the distal femur or proximal tibia. Several studies have validated the AUC guidelines as appropriate and in agreement with treatment recommendations [[11], [12], [13]]. CCI was also used in the present study to determine the burden of comorbidities for patients [14]. A score of 0 means that no comorbidities were found; the higher the score, the higher the predicted mortality rate is. Further information is described in the Supplementary Material.

**Figure 1:**
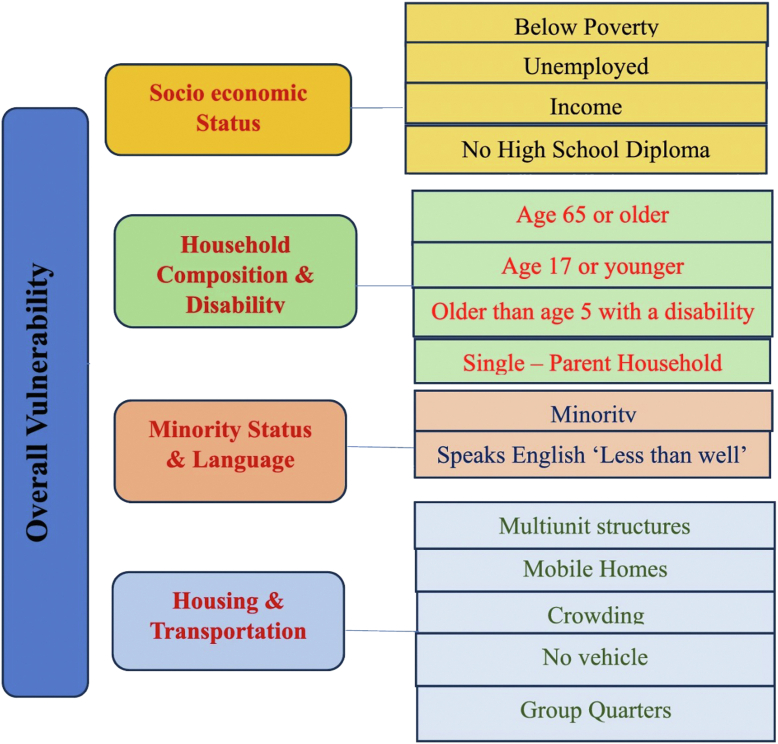
Variables used in Social Vulnerability Index calculation.

The SVI is designed to identify communities that may require additional support due to external stresses on human health, based on 4 themed scores: socioeconomic status, household characteristics, racial and ethnic minority status, and housing type and transportation [15]. The “household characteristics” theme includes factors such as the percentage of individuals aged 65 years or older, those aged 17 years or younger, civilians with disabilities, single-parent households, and those with limited English proficiency [15]. The “socioeconomic status” theme encompasses variables like income below 150% of the poverty level, unemployment rates, housing cost burden, lack of a high school diploma, and absence of health insurance [15]. The “housing type and transportation” theme includes multiunit structures and mobile homes, crowding, lack of vehicle access, and living in group quarters [15]. The “racial and ethnic minority status” theme covers the presence of NHW, Black or African American, American Indian or Alaska Native, Asian, and Native Hawaiian or Other Pacific Islander populations [[15], [16], [17]]. Figure 1 illustrates the distribution of variables used in the SVI calculation. The SVI ranges from 0.0 to 1.0, with higher scores indicating greater social vulnerability and a higher need for resources in each geographic area. The most recent SVI report from 2020 was accessed for this analysis. The SVI was then matched to the study population at the county level. A sensitivity analysis was conducted by matching SVI to the study population at the census tract level extracted from the ZIP code available in the dataset.

### Data analyses

The variables of interest included patient age, sex, race/ethnicity, insurance status, smoking status, CCI, and BMI. The addition of an independent variable for each of the 4 surgeons was also analyzed as additional potential covariates for TKA recommendation to control for potential confounders. Bivariate analyses were performed with Student's *t*-tests for continuous variables and *Chi*-square analyses for categorical variables to determine differences in surgical recommendation patient and surgeon factors. Logistic multivariable analyses tested covariates associated with TKA recommendations between patients recommended TKA by AUC guidelines only versus AUC and the surgeon. The outcomes of the multivariable analyses were odds ratios (ORs) and *P* values.

## Results

There were 402 consecutive patients who presented for management of knee OA to the clinics of 4 fellowship-trained arthroplasty surgeons. Of these, there were 314 patients (78.1%) rated “appropriate” for TKA by the AAOS guidelines (Table 1). Furthermore, there were 123 (39.2%) patients who were guideline-appropriate for TKA who received a surgeon recommendation for TKA (Table 2). Demographic data, BMI, and CCI by surgeon are provided in Table 2. For the patients considered “appropriate” for TKA, mean age was 68.66 years (range, 35-97 years) and race/ethnicity was 43.69% NHW, 38.51% Black, 2.59% Asian, and 10.36% Hispanic (Other) (Table 3).

**Table 3:** Bivariate and multivariable analyses analyzing covariates for surgeon recommendation of TKA.

Covariate	AUC recommendation only	AUC + surgeon recommendation	Bivariate *P* value	Multivariable OR	*P* value
Age	68.66 [35-97]	69.32 [38-91]	.239	0.988 [0.753-1.304]	.179
BMI	33.26 [19.58-67.43]	33.26 [19.58-67.43]	.007	0.963 [0.942-0.981]	.014
Charlson Comorbidity Index (CCI)	3.57 [0-11]	3.77 [0-11]	.091	0.524 [0.255-1.105]	.331
Surgeon					
Surgeon #1	38.51%	56.64%	.008	Used as reference variable.	Used as reference variable.
Surgeon #2	29.44%	19.47%	.001	0.434 [0.204-0.952]	.005
Surgeon #3	11.33%	3.54%	.001	0.209 [0.052-0.903]	.003
Surgeon #4	20.39%	20.35%	.067	0.795 [0.505-1.259]	.474
Gender					
Women	70.55%	34.51%	.079	0.635 [0.404-1.056]	.080
Men	29.45%	65.49%	.101	Used as reference variable.	Used as reference variable.
Insurance type					
Medicare	53.72%	52.21%	.319	Used as reference variable.	Used as reference variable.
Medicaid	11.97%	7.08%	.289	1.256 [0.804-1.756]	.362
Private insurance	33.33%	38.94%	.657	1.481 [0.953-2.203]	.685
Race/ethnicity					
NHW	43.69%	42.48%	.108	Used as reference variable.	Used as reference variable.
Black	38.51%	40.71%	.132	1.554 [0.904-2.308]	.114
Asian	2.59%	1.77%	.462	0.829 [0.504-1.358]	.790
Native American	0%	0%	1.000	0.394 [0.307-1.359]	1.000
Hispanic (Other)	10.36%	8.85%	.127	1.635 [0.955-2.503]	.292
Other	0.32%	0%	1.000	1.851 [0.948-2.441]	1.000
Declined	1.62%	1.77%	.657	2.703 [0.935-2.857]	.321
Unknown	1.62%	2.65%	.651	0.281 [0.055-0.409]	.015
Hispanic (White)	0.97%	0.88%	.423	1.854 [0.853-2.954]	.515
Smoking status					
Current	4.87%	2.65%	.011	Used as reference variable.	Used as reference variable.
Former	36.36%	37.17%	.090	3.475 [1.042-4.055]	.064
Never	58.77%	60.18%	.094	3.764 [1.066-4.067]	.053
SVI values					
Overall SVI	0.7556 [0.1152-0.8555]	0.6596 [0.1152-0.8555]	.284	0.705 [0.583-0.852]	.028
Socioeconomic status	0.7086 [0.1470-0.7756]	0.5127 [0.1470-0.7756]	.262	0.754 [0.628-0.892]	.034
Household characteristics	0.3971 [0.0668-0.7922]	0.2957 [0.0668-0.7922]	.357	0.803 [0.678-0.951]	.049
Racial and ethnic minorities	0.8932 [0.0302-0.9691]	0.8964 [0.0302-0.9691]	.153	0.951 [0.803-1.13]	.091
Housing type and transportation	0.8217 [0.0602-0.9930]	0.8269 [0.0602-0.9930]	.233	1.10 [0.902-1.35]	.124

AUC, Appropriate Use Criteria; BMI, body mass index; CCI, Charlson Comorbidity Index; NHW, Non-Hispanic White; OR, odds ratio; SVI, Social Vulnerability Index; TKA, total knee arthroplasty.

Surgeon #1 self-identified as Black, while Surgeons #2, #3, and #4 self-identified as NHW.

Mean and standard deviation reported for age, BMI, CCI, and SVI values.

Bivariate and multivariable analyses compared covariates among patients who received TKA recommendation from the AUC guidelines only to those who received TKA recommendation by surgeon and AUC recommendation, as seen in Table 3. Age and CCI showed no significant differences between the groups. BMI, however, was significantly associated with surgeon recommendations, with lower BMIs being more likely to receive a surgeon's endorsement (*P* = .007; OR: 0.963, *P* = .014). Variability among surgeons was evident, with Surgeons #2 and #3 significantly less likely to recommend TKA compared to Surgeon #1 (*P* = .001; OR: 0.434 and 0.209, respectively). Gender showed a trend, with women more likely to be recommended by AUC criteria alone and men more frequently receiving recommendations from both AUC and surgeons, although this was not evident in multivariable analysis (*P* = .080). Insurance type and race/ethnicity did not significantly impact recommendations, although slight variations were noted.

In analysis of SVI, patients with lower overall SVI values were more likely to receive a surgeon recommendation (0.7556 vs 0.6596, *P* = .284) (Table 3). Multivariable analysis confirmed that lower SVI values were independently associated with higher odds of surgeon recommendation (OR: 0.705, *P* = .028). Among the SVI subcategories, a lower score on the socioeconomic status subcategory was significantly associated with a higher likelihood of receiving a surgeon recommendation (OR: 0.754, *P* = .034). Additionally, lower scores in household characteristics (OR: 0.803, *P* = .049) were also significantly associated with increased odds of receiving a surgeon recommendation. The SVI components related to racial and ethnic minority status and housing type/transportation did not show significant associations in the multivariable analysis.

## Discussion

Mounting evidence has shown that demographic factors contribute to disparities in TKA utilization. Few studies have examined specific SDOH factors beyond race to explain this disparity. To our knowledge, this is the first study to explore whether the SVI, a composite measure of SDOH, demonstrates differences in patients deemed appropriate for TKA by both AAOS guidelines and individual surgeon recommendations. Our stratification of SVI into socioeconomic status, household characteristics, racial/ethnic minority status, and housing/transportation further pinpoints which components of social vulnerability influence these disparities. Additionally, categorization of data by surgeon highlights potential behavioral factors, such as provider relationships and biases. Unlike previous studies that focused on patients undergoing TKA, our approach captures those eligible for surgery per physician recommendation, offering a broader perspective on potential disparities.

Our findings suggest that patients deemed appropriate for TKA by surgeons exhibit lower overall social vulnerability compared to those meeting only AAOS guidelines. This difference may reflect the influence of qualitative factors, such as socioeconomic status and household characteristics, that surgeons assess during patient interactions. These factors, often inferred from patient disclosures or chart data (eg, insurance status, employment, ZIP code), may shape surgeons' perceptions of a patient's suitability for surgery and their ability to navigate postoperative recovery. For example, unsuitable housing environments may lead some surgeons to deem patients ineligible for TKA recovery. However, this consideration was observed more prominently in 2 of the 4 surgeons studied, indicating that surgeon-specific decision-making warrants further exploration.

Disparities between surgeons in their likelihood of recommending TKA also highlight the complexity of this issue. Two surgeons were significantly less likely to recommend TKA than Surgeon #1, raising questions about how individual background, training, and implicit biases influence decision-making. The multifaceted nature of these factors—spanning socioeconomic, cultural, political, and behavioral domains—complicates efforts to identify their root causes and requires additional investigation.

Several limitations of this study merit discussion. First, while we examined the 4 overarching SVI components, further stratification into more detailed demographic or social determinants was not feasible. Second, the study population was drawn from an academic practice at a tertiary care hospital serving the greater Chicagoland area and surrounding states, which may limit generalizability. Additionally, the study's enrollment period and patient volume were limited and selection bias may have influenced the data, as these surgeons likely see a higher volume of patients in clinical practice. Furthermore, an additional avenue for further exploration would be to investigate how individual surgeons assess and incorporate socioeconomic variables in their decision-making process when recommending TKA. Factors such as how surgeons approach history taking, review demographic information, and account for inherent physical biases could all play significant roles in influencing their clinical decisions. Additionally, it would be valuable to explore whether surgeons with more experience or specialized training exhibit different patterns in considering socioeconomic factors compared to their less experienced counterparts. Understanding these influences could offer important insights into the mechanisms behind the disparities in TKA utilization and guide strategies for reducing biases in clinical decision-making. Future studies with larger patient cohorts and extended enrollment periods may help mitigate this limitation and provide greater insight into these disparities.

Despite these limitations, our findings suggest that disparities in TKA utilization are influenced by specific aspects of social vulnerability, particularly socioeconomic status and household characteristics. The observed association between SVI and TKA appropriateness highlights SVI as a potentially valuable tool for identifying SDOHs that act as barriers to surgical care. Targeted mitigation strategies addressing these disparities warrant further evaluation to promote equitable surgical care access.

## Conclusions

Patients for whom the AAOS AUC classified TKA as “appropriate” but did not receive a surgeon recommendation for surgery had more vulnerable SVI scores. The interaction between social vulnerability and decision-making in clinic may be one driver of underutilization of TKA. Further evaluation is warranted to mitigate the biases that influence surgical decision-making.

## Conflicts of interest

Adam Edelstein is the editorial board member of Arthroplasty Today. All other authors declare no potential conflicts of interest.

For full disclosure statements refer to https://doi.org/10.1016/j.artd.2025.101702.

## CRediT authorship contribution statement

**Manasa Pagadala:** Writing – review & editing, Writing – original draft, Visualization, Validation, Software, Investigation, Formal analysis, Data curation. **Linda I. Suleiman:** Supervision, Resources, Project administration, Methodology, Investigation, Data curation, Conceptualization. **Rachel Bergman:** Writing – review & editing, Writing – original draft, Investigation. **T. Jacob Selph:** Writing – review & editing, Writing – original draft, Visualization, Investigation, Data curation. **Patricia Franklin:** Supervision, Resources, Project administration, Methodology, Investigation, Data curation, Conceptualization. **Adam I. Edelstein:** Supervision, Resources, Project administration, Methodology, Investigation, Data curation, Conceptualization.

## Appendix

The American Academy of Orthopaedic Surgeons employs the RAND/University of California, Los Angeles Appropriateness Method, a research method developed by the RAND Corporation and the University of California, Los Angeles to assess the appropriateness of medical and surgical procedures. It uses available scientific literature in conjunction with clinical expertise of physicians of multiple specialties to develop clinical scenarios. Expert panels comprised of representatives from these medical specialties evaluate these scenarios and determine the appropriateness of clinical recommendations by rating clinical scenarios as “appropriate,” “may be appropriate,” or “rarely appropriate.” An “appropriate” procedure is defined as one for which the expected health benefits exceed the expected negative consequences by a sufficiently wide margin [2].

The specific Appropriate Use Criteria (AUC) “Osteoarthritis of the Knee: Surgical Management,” published in 2016, was devised by an expert voting panel which considered 864 unique clinical scenarios to determine ratings for each. The AUC is an indication profile comprised of 8 observable patient parameters: (1) function-limiting pain, (2) range of motion extension/flexion, (3) functional instability, (4) pattern of arthritic involvement, (5) imaging, (6) limb alignment, (7) mechanical symptoms, and (8) age. This AUC is accompanied by several assumptions that surgeons should consider prior to utilization to ensure that the criteria are employed in the correct clinical setting (ie, adults who have been diagnosed with primary osteoarthritis of the knee who have failed nonoperative treatments). A web-based application can be found at www.orthoguidelines.org/auc [9,10] to help clinicians develop a plan for managing knee osteoarthritis. These guidelines serve as to address surgeon questions regarding the use of total knee arthroplasty, unicompartmental knee arthroplasty, and local osteotomy at the level of the distal femur or proximal tibia.

The Charlson Comorbidity Index predicts the mortality for a patient who may have a range of comorbidities, such as heart disease, AIDS (Acquired Immunodeficiency Syndrome), or cancer (considering a total of 17 categories). A score of 0 means that no comorbidities were found; the higher the score, the higher the predicted mortality rate is. For a physician, this score is helpful in deciding how aggressively to treat a condition. Charlson Comorbidity Index is one of the most widely used scoring system for comorbidities.

## Appendix A Supplementary data

Conflict of Interest Statement for Bergman

Conflict of Interest Statement for Edelstein

Conflict of Interest Statement for Franklin

Conflict of Interest Statement for Pagadala

Conflict of Interest Statement for Selph

Conflict of Interest Statement for Suleiman
