# Supplementary material for: Evaluating the Disparate Use of Knee Arthroplasty Among Minorities Using Social Vulnerability Index
Source: Arthroplast Today. 2025 Jun 9;33:101702. doi: 10.1016/j.artd.2025.101702 (PMC12179742; doi:10.1016/j.artd.2025.101702)
Supplement: Conflict of Interest Statement for Suleiman [file mmc6.docx]

# INDIVIDUAL CONFLICT OF INTEREST STATEMENT

***American Association of Hip and Knee Surgeons***

(Adopted from the American Academy of Orthopaedic Surgeons disclosure statement)

The following form **must be filled out completely and submitted by each author (example, 6 authors, 6 forms).**

**All items require a response. If there is no relevant disclosure for a given item, enter "*None*.”**

**Manuscript Title: Evaluating the Disparate Use of Knee Arthroplasty among Minorities using Social Vulnerability Index**

1. Royalties from a company or supplier (The following conflicts were disclosed): None

2. Speakers bureau/paid presentations for a company or supplier (The following conflicts were disclosed): None

3A. Paid employee for a company or supplier (The following conflicts were disclosed): None

3B. Paid consultant for a company or supplier (The following conflicts were disclosed): None

3C. Unpaid consultants for a company or supplier (The following conflicts were disclosed) : None

4. Stock or stock options in a company or supplier (The following conflicts were disclosed) : None

5. Research support from a company or supplier as a Principal Investigator (The following conflicts were disclosed) : None

6. Other financial or material support from a company or supplier (The following conflicts were disclosed) : None

7. Royalties, financial or material support from publishers (The following conflicts were disclosed) : None

8. Medical/Orthopaedic publications editorial/governing board (The following conflicts were disclosed) : None

9. Board member/committee appointments for a society (The following conflicts were disclosed) : None

**Each author must sign AND print or type his/her name, date and submit a separate form**

In addition, one BLINDED Conflict of Interest form (no author names used) should be submitted per manuscript with all author disclosures.

Linda I. Suleiman Linda Suleiman (electronic) 3/29/24

Author Name (Print or Type) Author Signature Date
